# Oral Rehabilitation of Severely Atrophic Alveolar Ridge Using Sinus Augmentation and Ridge Split Technique: A Case Report

**DOI:** 10.7759/cureus.114450

**Published:** 2026-08-12

**Authors:** Rashmita Majhi, Reni E Mammen, Lokanath Garhnayak, Rakesh K Majhi, Swapnil Parlani

**Affiliations:** 1 Department of Prosthodontics and Crown and Bridge, Srirama Chandra Bhanja (SCB) Dental College and Hospital, Cuttack, IND; 2 Department of Prosthodontics and Crown and Bridge, People's College of Dental Sciences and Research Centre, Bhopal, IND; 3 Department of Biological Sciences and Bioengineering, Indian Institute of Technology Kanpur, Kanpur, IND

**Keywords:** dental implants, implant prostheses, ridge splitting, severely atrophied ridge, sinus augmentation

## Abstract

Dental implant placement requires adequate bone volume. Sinus augmentation and ridge split technique enhance bone volume in a severely atrophied alveolar ridge. This case report describes the rehabilitation of a patient with severe posterior maxillary atrophy (residual bone height <2.5 mm) and a knife-edge posterior mandibular ridge using lateral sinus floor elevation and ridge split technique, respectively, followed by implant placement. Given the limited high-level evidence on the combined management of these complex anatomical deficiencies and their long-term implant outcomes, this report highlights the clinical feasibility and successful rehabilitation achieved with these site-specific augmentation procedures. Following this, dental rehabilitation was performed using an implant-supported FP3 prosthesis, precision attachments, tooth-supported bridges, and crowns. At the two-year follow-up, all implants were stable without mobility or complications. Clinical examination showed healthy peri-implant tissues, acceptable probing depths, absence of bleeding on probing and suppuration. Radiographs demonstrated stable marginal bone levels and maintained grafted site integrity. This article highlights successful alveolar ridge augmentation in a patient with severely resorbed ridges with height ≤2.5 mm. Implant-supported prostheses yielded satisfactory results during the two-year follow-up period.

## Introduction

Implant-supported prostheses are a widely recognized treatment modality. The quantity and quality of the supporting alveolar bone at the intended site determine the stability and success of implant treatment [1]. Distraction osteogenesis, sinus lift with bone grafts, inferior alveolar nerve relocation, and guided bone regeneration (GBR) have been reported to enhance bone volume [2,3]. Implant placement in the atrophic posterior maxilla is difficult due to insufficient posterior alveolus and increased maxillary sinus pneumatization [4]. In these situations, sinus augmentation is the most reliable technique for placing endosseous implants, and the commonly used approaches for sinus lift surgery are the crestal route and the lateral window technique [1,5,6]. Maxillary sinus augmentation and implant insertion can be performed using three surgical approaches: two-step lateral sinus augmentation, single-step lateral sinus augmentation, or a one-step crestal approach with simultaneous implant placement [7]. Implant placement is carried out following four to six months of bone grafting if less than 5 mm of residual bone height is present (two-step technique). After four to six months of healing following implant placement, abutments are subsequently installed [4]. Inorganic bovine bone minerals and autologous bone have similar implant survival rates and are most often used for bone augmentation [8,9]. Bio-Oss (Geistlich, Switzerland) is sterilized, deproteinized bovine bone made up of carbonate apatite crystals with low calcium content, measuring roughly 10 nm in size, and its chemical and physical characteristics are similar to those of human bone [10].

Ridge splitting is another bone augmentation technique to enlarge the ridge horizontally by widening the cortical plates of a narrow edentulous ridge (>3.5 mm). For a successful outcome, at least 1-1.5 mm of bone should be present on the buccal and lingual/palatal sides of the implant(s) [11,12]. This case report describes the staged rehabilitation of severe horizontal and vertical alveolar ridge deficiencies using two site-specific augmentation approaches: sinus augmentation with delayed implant placement in the posterior maxilla with ≤2.5 mm of residual alveolar bone, and ridge splitting with simultaneous implant placement in the narrow posterior mandible. The novelty of this report lies in the combined application of these techniques within the same patient to manage complex ridge deficiencies at different anatomical sites, with clinical outcomes documented over a two-year follow-up after prosthetic loading.

## Case presentation

A 45-year-old woman presented with the chief complaint of difficulty in mastication and unaesthetic appearance. She had no relevant medical history, with no reported diabetes, tobacco use, antiresorptive medication use, or sinus disease. 

Clinical findings and diagnostic assessment

**Figure 1:**
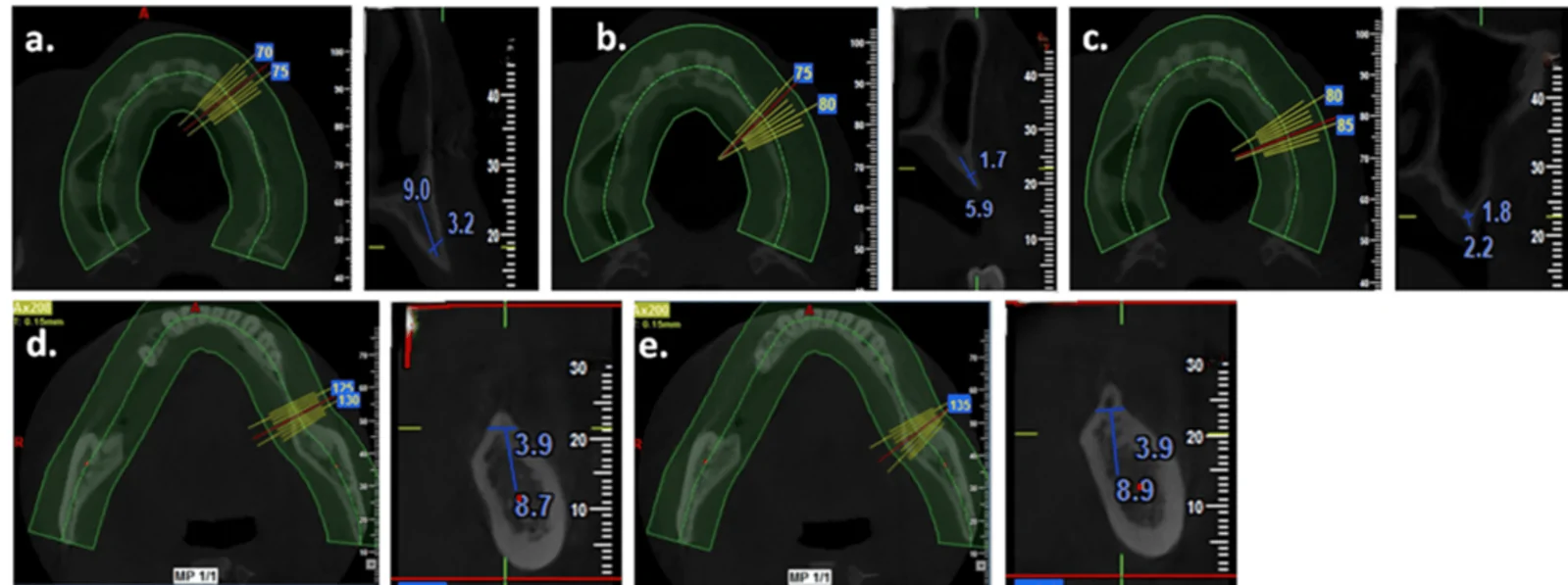
Preoperative cone beam computed tomography assessment of bone dimensions at the proposed implant sites. Preoperative cone beam computed tomography of (a) implant site 23, (b) implant site 25, (c) implant site 27, (d) implant site 36, and (e) implant site 37.

Clinical examination revealed maxillary edentulous space at teeth# 13, 15, 16, 23, 24, 25, 26, 27 and mandibular edentulous space at teeth# 36, 37, 44, 46, 47. Carious-exposed teeth at 11, 12, 14, 22, 34, 35, 45 and supra-erupted teeth at 17 were present; therefore, the patient underwent endodontic treatment. The patient was not interested in removable partial dentures. Preoperative imaging and cone beam computed tomography (CBCT) revealed the bone dimensions at proposed implant sites as follows: site 27: 2.2 mm height, 1.8 mm width; site 25: 5.9 mm height, 1.7 mm width; site 23: 9.0 mm height, 3.2 mm width; site 36: 8.7 mm height, 3.9 mm width; site 37: 8.9 mm height, 3.9 mm width (Figure 1). Thus, bone augmentation techniques were required to support the implants for a fixed prosthesis. The proposed treatment comprised implant placement at 36 and 37 using the ridge split technique and posterior left maxillary sinus augmentation using the lateral two-step technique, followed by implant placement at 23, 25, and 27 sites six months later. Thereafter, these implant sites were rehabilitated by implant-supported prostheses. Tooth-supported fixed partial denture (FPD) at 11, 12, 13, 14, 15, 16, 17 and porcelain-fused metal (PFM) crowns at 22, 34, 35 were planned. PFM bridge for 43, 44, 45, with a precision attachment for replacing 46, 47, was also designed.

Therapeutic intervention

A direct lateral window sinus lift technique was used to achieve sinus augmentation at the proposed maxillary implant sites to increase ridge width and height [8]. Briefly, midcrestal and vertical incisions were made, and a full-thickness flap was raised above the mucogingival junction. On the sinus lateral wall, a window was prepared and infractured, and the Schneiderian membrane was gently raised. Following this, the intended implant sites were packed with an inorganic bovine bone graft (Bio-Oss). A sterile, trimmed bioresorbable collagen membrane (Healiguide; Advanced Biotech, Chennai, India) was placed over the graft to promote GBR. Flaps were sutured first with horizontal mattress sutures, followed by single interrupted sutures (Ethicon Mersilk Black Braided Nonabsorbable Suture; Ethicon, USA) to close flap edges. Sutures were removed after 10 days.

**Figure 2:**
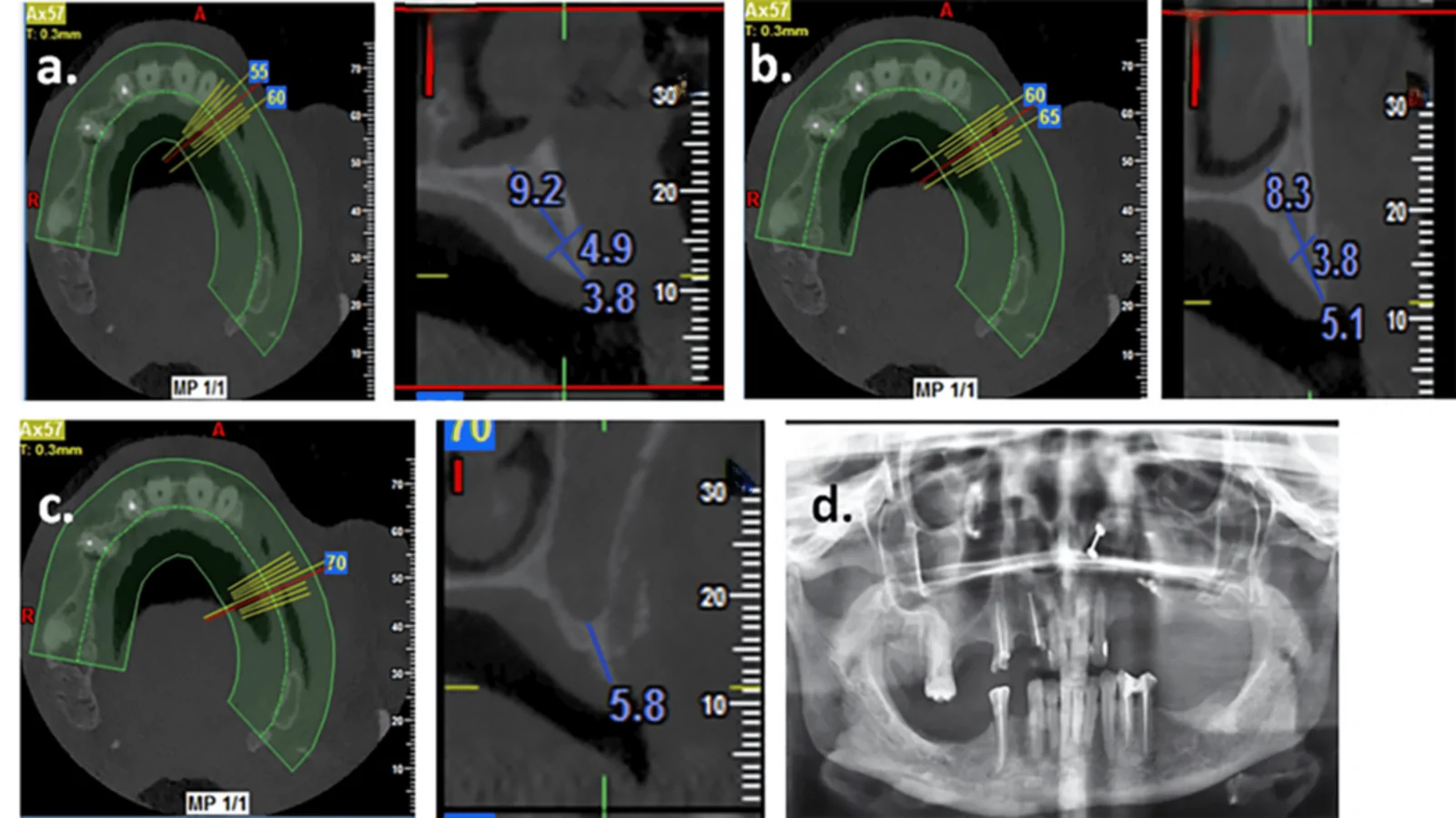
Postoperative assessment for implant placement at the implant sites. a) CBCT of region 23. (b) CBCT of region 25. (c) CBCT of region 27. (d) OPG X-ray image after sinus augmentation. CBCT: cone beam computed tomography; OPG: orthopantomogram

**Figure 3:**
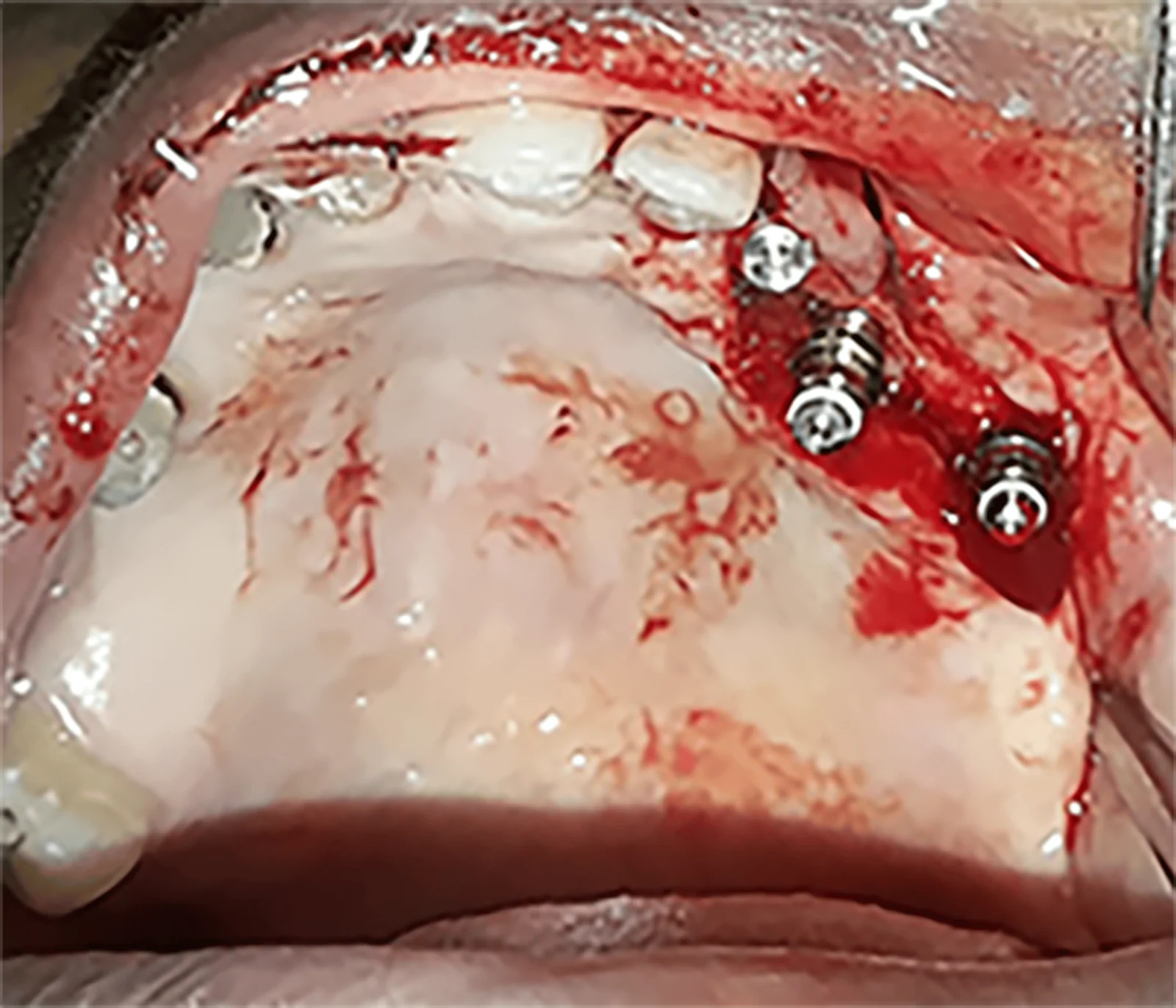
Implants placed at 23, 25, and 27 regions after sinus augmentation.

After the six-month healing period, orthopantomogram (OPG) and CBCT images showed a significant increase in bone volume with a vertical height of 13 mm, 12.1 mm, and 5.8 mm at implant sites 23, 25, and 27, respectively. The horizontal width increased to 4.9 mm, 5.1 mm, and 5.8 mm at implant sites 23, 25, and 27, respectively (Figure 2a-2d). Subsequently, three endosteal Osstem implants 3.0 × 10 mm, 3.5 × 10 mm, and 4.0 × 10 mm (Osstem Implant Co. Ltd., South Korea) were placed at 23, 25, and 27 regions, respectively, following the standard surgical protocol (Figure 3). The implants were kept submerged to promote healing and bone integration around them.

**Figure 4:**
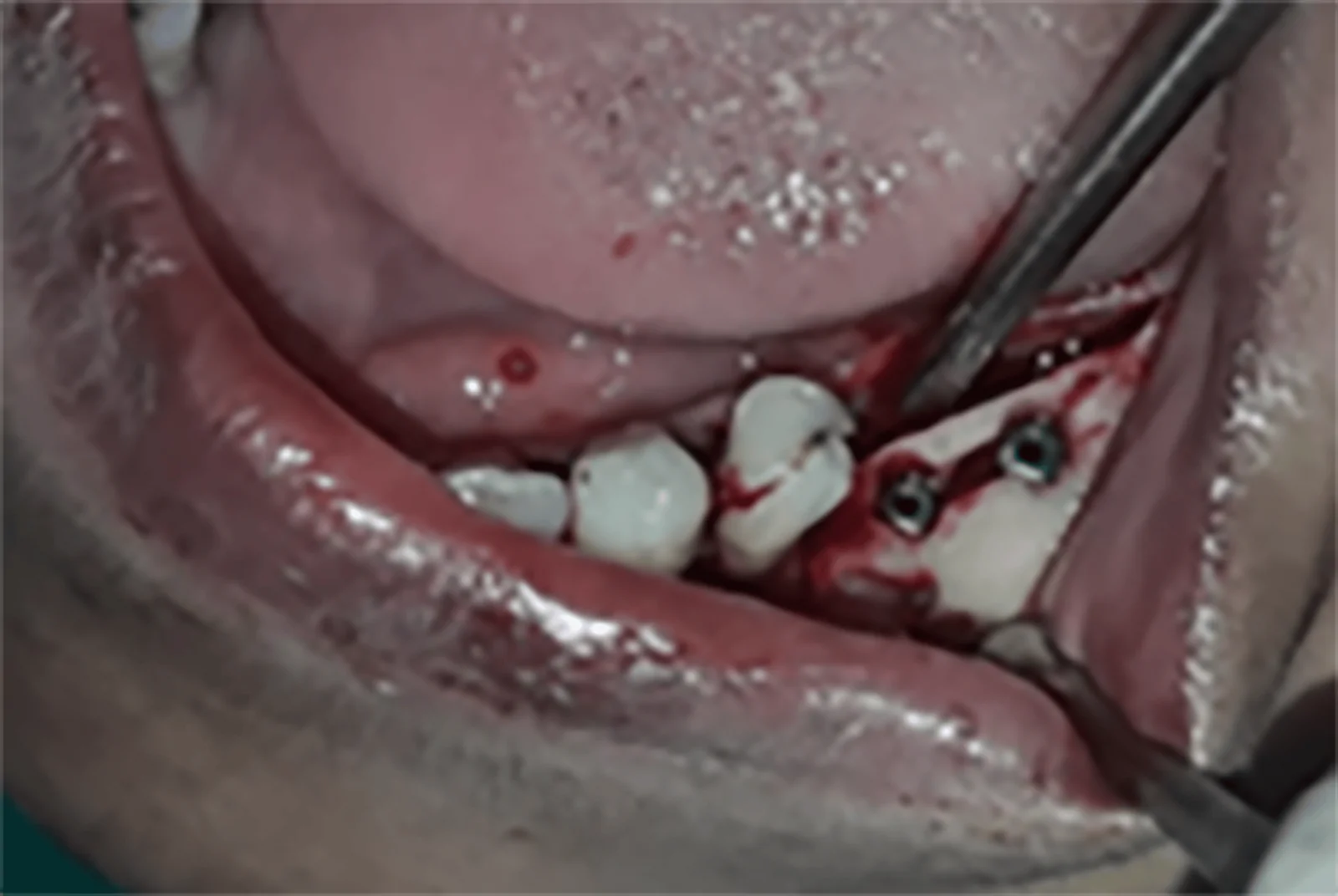
Implants placed at 36 and 37 regions by ridge splitting.

**Figure 5:**
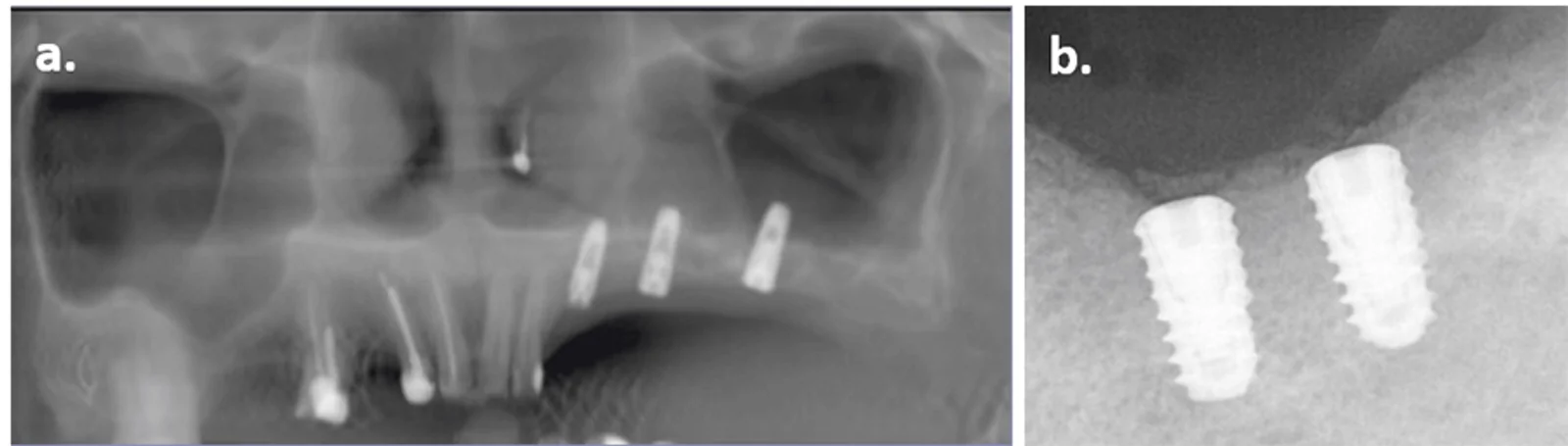
Postoperative X-ray images taken after six months show radiographically stable implants. (a) Orthopantomogram (OPG) image of maxillary implants placed at 23, 25, and 27 sites. (b) Radiovisiography image of mandibular implants placed at 36 and 37 sites.

In another appointment, ridge splitting was performed on the narrow left mandibular ridge as previously reported [11]. Access to the surgical site was accomplished by crestal and vertical incisions followed by reflection of a full-thickness mucoperiosteal flap. Crestal horizontal corticotomy was started 1 to 2 mm away from the adjacent tooth using ultrasonic instruments. Two vertical corticotomies were created on the buccal cortical plate to regulate mandibular bone flexibility. Buccal and lingual cortical bone was gradually expanded using a motorized bone expander (MCTBIO bone expander kit, South Korea). Implant sites were prepared using a twist drill, and two 4.0 × 10 mm implants were placed. Each was positioned in 36,37 regions, respectively, in the created space (Figure 4). Then, the defects were filled with inorganic bovine bone grafts (Bio-Oss), covered by a resorbable membrane (Healiguide), and sutures were placed (Ethicon Mersilk Black Braided Nonabsorbable Suture). Primary stability above 35 Ncm was achieved in all implants. Postoperative X-ray images taken after a six-month healing period showed sufficient bone-implant contact and no marginal bone loss (Figure 5a, 5b).

Prosthetic phase

**Figure 6:**
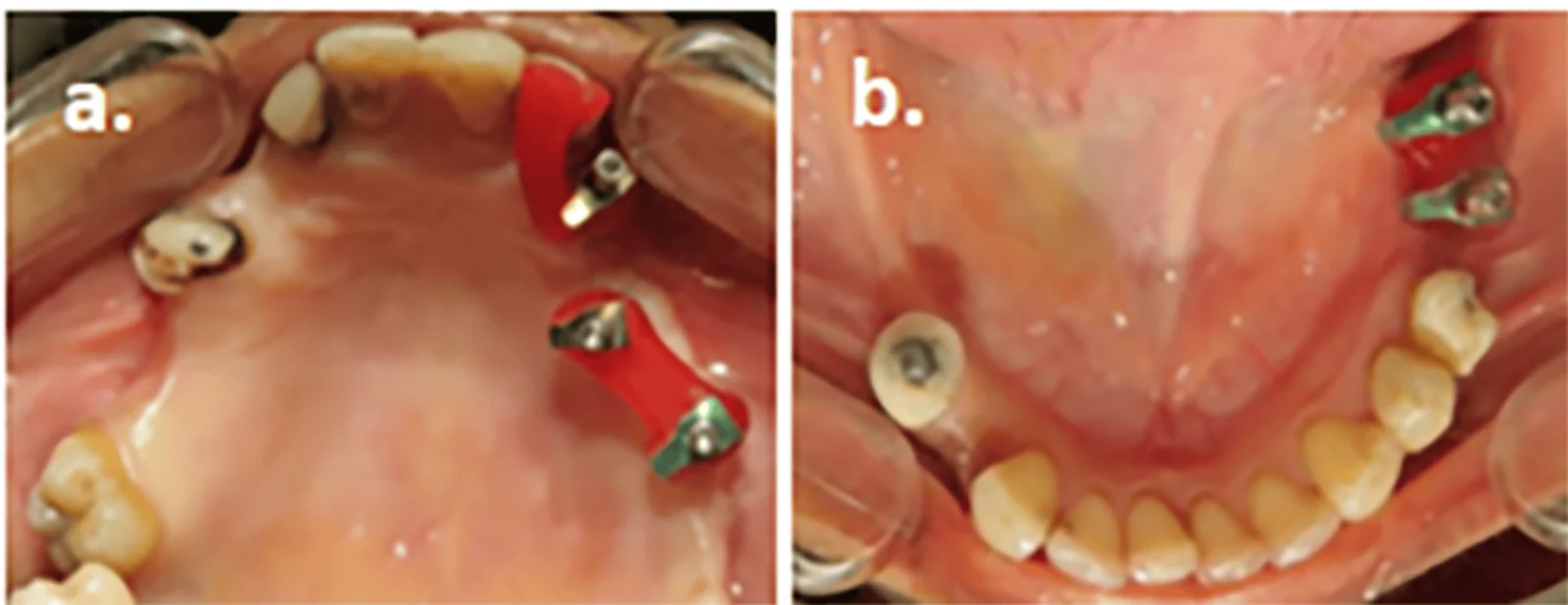
(a) Jig trial of maxillary implants. (b) Jig trial of mandibular implants.

**Figure 7:**
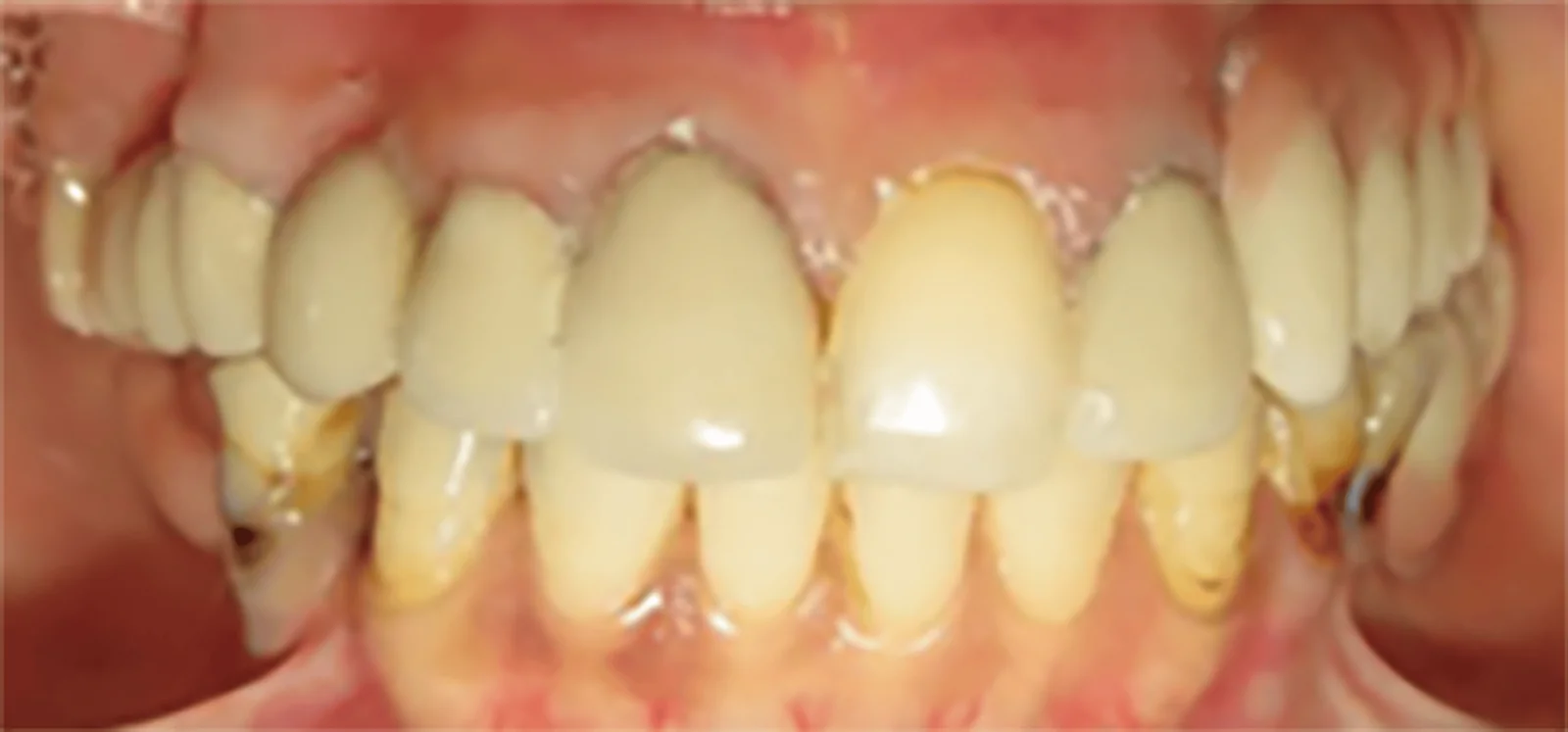
Cementation of final maxillary prostheses and mandibular screw-retained prostheses.

After six months of the osseointegration phase, stage II surgery was performed, and healing abutments were positioned. Open-tray impressions were taken using polyvinyl siloxane (Zhermack Elite HD, Italy). The implants exhibited significant angulation discrepancies of around 18 degrees that precluded a common prosthetic path of insertion with stock components. Therefore, customized castable abutments were fabricated to correct the prosthetic path, optimize the emergence profile, and facilitate passive seating of the definitive framework. A verification jig trial was performed, and passive fit was verified clinically and confirmed radiographically (Figure 6). Metal post and composite fillings were placed at teeth# 12, 14, 34, 35. Thereafter, final impressions of prepared teeth at 11, 12, 14, 17, 22 were taken with polyvinyl siloxane (Zhermack Elite HD), and provisional restorations were fabricated. The occlusal plane for the provisional maxillary prosthesis was established, and the shade was selected. Thereafter, maxillomandibular relation was obtained, and interocclusal records were registered using bite registration wax (MAARC, India). Following this, the metal framework trial, occlusion and proximal contacts were checked in the pre-glaze trial prosthesis. The definitive tooth-supported FPD replacing teeth 11-17 and 22 was cemented using glass ionomer luting cement (GC Gold Label; Tokyo, Japan). The implant-supported cement-retained FP3 prosthesis replacing teeth 23-27 was also cemented, and excess cement was carefully removed from the abutment margins. FP3 prosthesis replacing teeth 36, 37 was screwed onto abutments (Figure 7).

**Figure 8:**
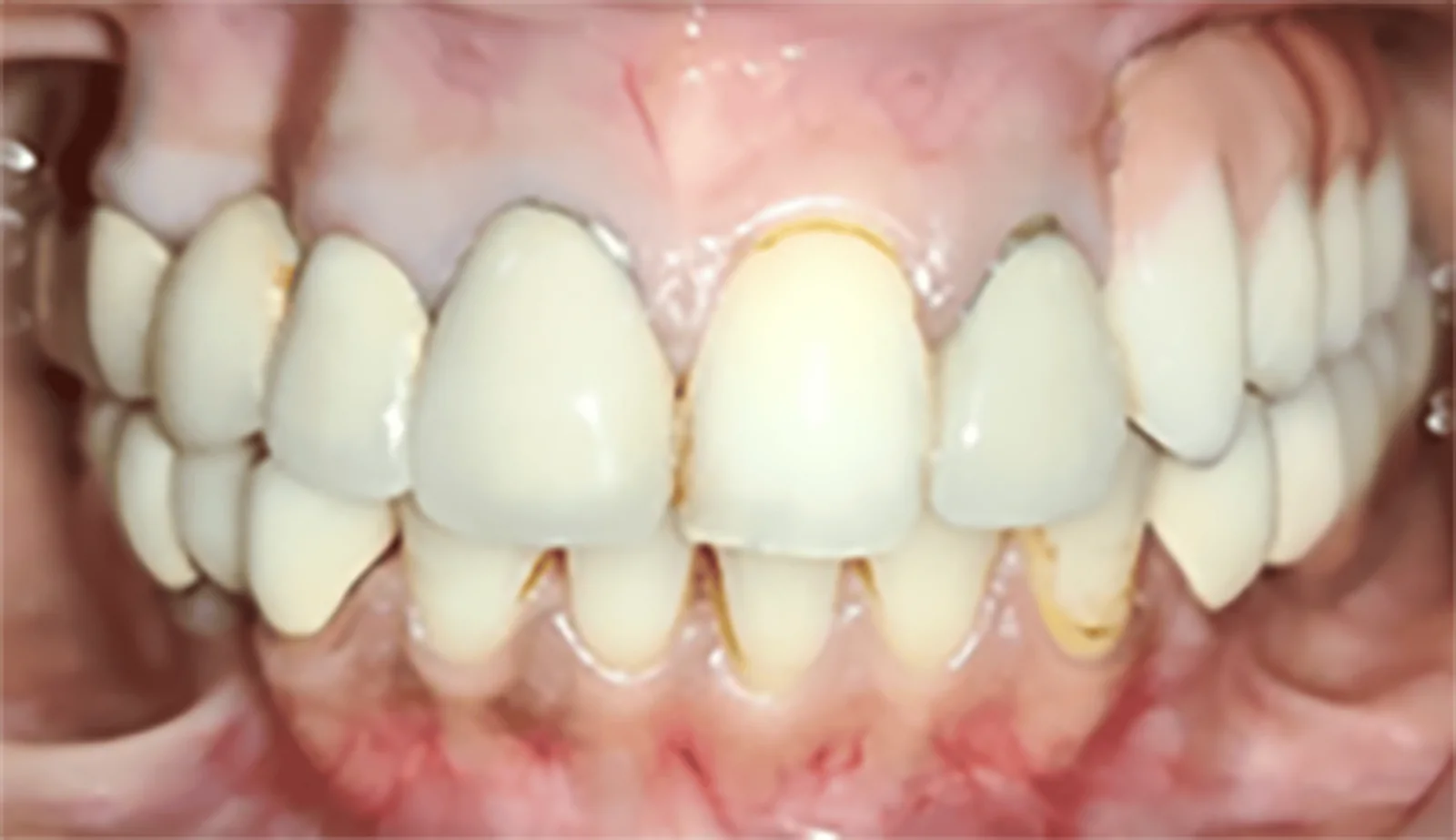
Postoperative image after cementation of final prostheses.

Thereafter, final impressions of prepared teeth at 34, 35, 43, 45 were taken using polyvinyl siloxane (Zhermack Elite HD). A trial of a metal coping for 34, 35 and a precision attachment (OT Unilateral, Rhein 83, USA) on a tooth-supported PFM bridge at 43, 44, 45 was done. The precision attachment consisted of a male section having a vertical strut extending through the attachment's base to provide enhanced retention, lateral stability, and distal support to the prostheses replacing 46, 47. The female section consisted of a one-piece castable housing covering the male section. Finally, the fabricated PFM crowns for 34, 35 and PFM bridge for 43, 44, 45 were cemented using GIC luting cement (GC Gold label) and precision attachment replacing 46,47 was placed. Occlusion was adjusted to establish mutually protected occlusion with even centric contacts and to eliminate premature interferences during excursive movements. The prosthetic design incorporated embrasure spaces and a modified tissue surface to facilitate oral hygiene (Figure 8).

Follow-up and outcomes

**Figure 9:**
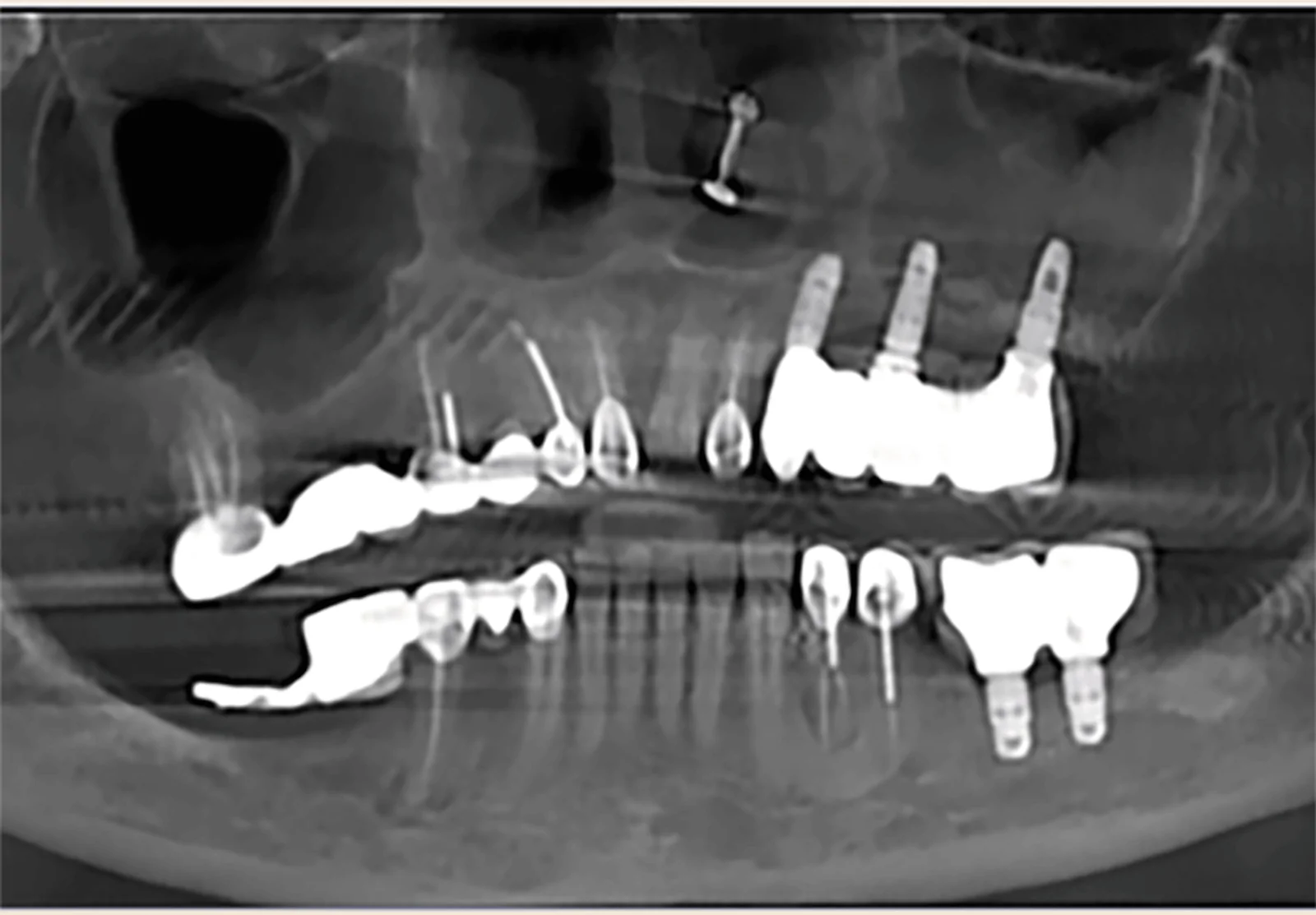
OPG X-ray taken one month after prosthesis delivery. OPG: orthopantomogram

**Figure 10:**
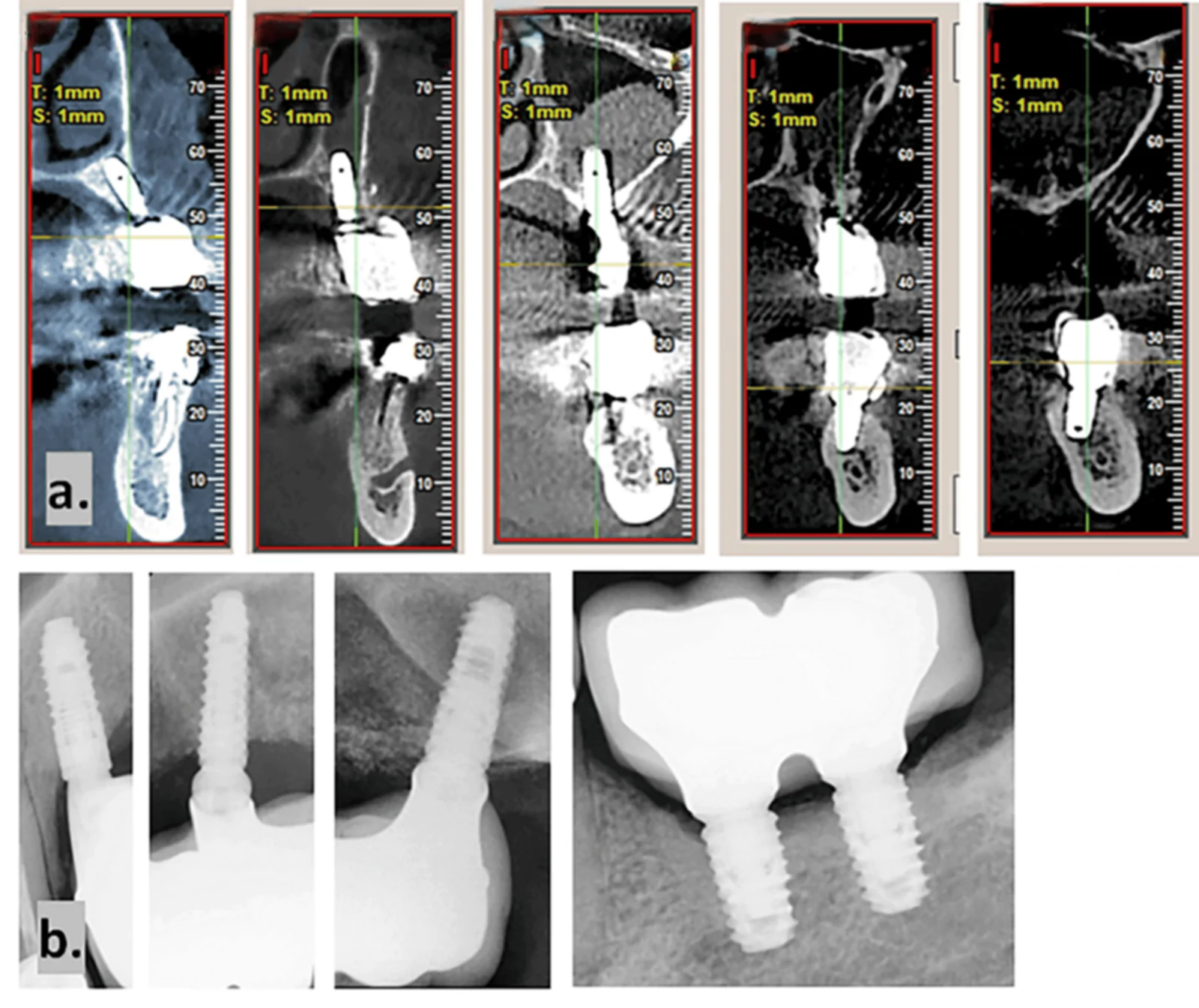
(a) Cone beam CT of implant sites 23, 25, 27, 36, and 37, respectively, taken at the two-year follow-up period after prosthesis delivery. (b) RVG of implant sites 23, 25, 27, 36, and 37, respectively, taken at the two-year follow-up period after prosthesis delivery. CT: computed tomography; RVG: radiovisiography

A postoperative OPG was obtained one month after prosthesis delivery (Figure 9). The patient was followed up to evaluate clinical implant stability, function, and marginal bone loss. CBCT scans and RVG of all implant-supported prostheses after prosthesis loading were obtained at two-year follow-up (Figure 10). These showed minor crestal bone loss, expected during the initial years of functional loading. The implants were clinically stable, and functional with healthy peri-implant soft tissues. The patient was satisfied in both aesthetic and functional aspects of the prostheses.

## Discussion

Sinus floor augmentation and GBR have emerged as an outstanding therapy for enhancing alveolar ridge dimensions in patients with atrophied posterior maxillae [8]. Besides residual bone height being an important criterion, bone substitute materials, implant materials, and surgical procedures contribute to the success of the implantation [13].

Most studies have reported the use of allograft, autograft, or a mixture of autologous and inorganic bovine bone as an effective means of achieving adequate new bone formation [8,14,15]. However, Sartori et al. demonstrated that inorganic bovine bone graft (Bio-Oss) is also an ideal graft material for increasing bone volume in maxillary sinus augmentation [16]. Very few studies have reported bone gain and peri-implant stability using inorganic bovine bone graft (Bio-Oss) for sinus augmentation and GBR in a severely resorbed maxillary posterior ridge [14,15]. The choice to proceed with a two-stage implant placement was made due to insufficient residual crestal bone, which compromised the ability to achieve primary implant stability.

Thus, this case report presents clinical outcomes of sinus floor augmentation using only inorganic bovine bone graft (Bio-Oss) and delayed implant placement in a severely atrophied posterior maxillary ridge with a ridge height <2.5 mm. OPG and CBCT scans taken six months after maxillary implant placement showed a significant increase in bone volume at the intended implant sites, allowing placement of endosseous implants. The clinical and radiographic outcomes demonstrated radiopaque bone fill, consistent with the findings of previous studies [8,16]. However, the extent of new bone formation and the rate of graft resorption could not be definitively assessed because histologic or histomorphometric analyses were not performed. 

The lateral ridge expansion procedure is quite effective for horizontal ridge augmentation in narrow posterior mandibular ridges having a thin cortex and low bone quality [15,17]. In this patient, an initial ridge width of approximately 3.9 mm was considered suitable for ridge splitting based on the clinical and radiographic assessment of the cortical morphology. However, ridge splitting in the mandible carries recognized risks, including cortical plate fracture, dehiscence, and neurosensory injury, necessitating careful case selection and surgical technique. Following this technique, the healing period in this case was reduced to six months in the mandible. Thus, the ridge-splitting procedure performed in our patient resulted in successful outcomes, including increased bone width and osseointegration.

Rehabilitation of the distal extension was challenging because the patient declined additional implant surgery and conventional removable prosthesis options after counseling. As the absence of a distal abutment precluded a conventional FPD, a removable tooth-supported prosthesis retained by an extracoronal precision attachment was selected. The patient was informed of the maintenance requirements, and this treatment option was accepted. Furthermore, the patient was recalled, and a radiological follow-up two years later showed minimal crestal bone resorption at the implant sites with no complications. All implants were stable, and the patient was satisfied with the aesthetics and function of her dental prostheses.

In summary, this case illustrates the application of GBR and maxillary sinus augmentation using an inorganic bovine bone graft (Bio-Oss) with resorbable barrier membranes for maxillary reconstruction, with favorable clinical and radiographic outcomes. However, the findings are limited by the short-term evaluation of implant survival. To strengthen the evidence base, further long-term studies are warranted to assess the performance of biomaterials and explore alternative techniques to achieve substantial bone regeneration and minimize crestal bone resorption in cases of severe maxillary ridge atrophy.

## Conclusions

Within the limitations of this study, implant-supported oral rehabilitation in severely resorbed ridges appears clinically feasible using GBR for horizontal and/or vertical bone augmentation, including sinus augmentation and ridge splitting with inorganic bovine bone grafts. In the present case, implants placed in a residual bone height of less than 2.5 mm remained functional during the two-year follow-up period after prosthetic loading. Additionally, transverse ridge expansion may be a suitable alternative to more invasive bone augmentation procedures in carefully selected cases, depending on ridge morphology, cortical bone thickness, and operator experience. Thus, augmentation of deficient alveolar ridges remains a crucial component of implant therapy, requiring comprehensive training in both hard and soft tissue management to facilitate functional and aesthetic restorations, as demonstrated in this case report.
